# Effect of Selected Anionic and Cationic Drugs Affecting the Central Nervous System on Electrical Properties of Phosphatidylcholine Liposomes: Experiment and Theory

**DOI:** 10.3390/ijms22052270

**Published:** 2021-02-25

**Authors:** Joanna Kotyńska, Monika Naumowicz

**Affiliations:** Department of Physical Chemistry, Faculty of Chemistry, University of Bialystok, K. Ciolkowskiego 1K, 15-245 Bialystok, Poland; monikan@uwb.edu.pl

**Keywords:** perphenazine, barbituric acid, liposomes, charged drugs, pH, surface charge, adsorption equilibria, electrophoretic light scattering

## Abstract

Interactions between phospholipid membranes and selected drugs affecting the central nervous system (CNS) were investigated. Small, unilamellar liposomes were used as biomimetic cell membrane models. Microelectrophoretic experiments on two-component liposomes were performed using the electrophoretic light scattering technique (ELS). The effect of both positively (perphenazine, PF) and negatively (barbituric acid, BA) charged drugs on zwitterionic L-α-phosphatidylcholine (PC) membranes were analyzed. Experimental membrane surface charge density (δ) data were determined as a function of pH. Quantitative descriptions of the adsorption equilibria formed due to the binding of solution ions to analyzed two-component membranes are presented. Binding constants of the solution ions with perphenazine and barbituric acid-modified membranes were determined. The results of our research show that both charged drugs change surface charge density values of phosphatidylcholine membranes. It can be concluded that perphenazine and barbituric acid are located near the membrane surface, interacting electrostatically with phosphatidylcholine polar heads.

## 1. Introduction

Biological membranes constitute the existence of each living cell, not only because they separate the cell (in the case of external membranes—plasma) or cell organelles (in the case of internal membranes) from the environment and through a series of life processes occurring with their participation. Natural cell membranes present a great complexity of structure (comprising various constituents such as lipids, carbohydrates, proteins), cross-connections, and functionality; therefore, simplified artificial membrane systems have been developed [1,2]. Up-and-coming model membrane systems which mimic the fundamental structural and functional properties of natural membranes are liposomes, particularly those formed from phosphatidylcholine—one of the predominant lipids in the cellular membrane [3,4]. Those biomimetic systems enable the study of drug–membrane interactions under defined and controlled conditions. Liposomes are highly suitable for determining binding parameters and allow the use of various techniques, such as microelectrophoresis [5,6,7].

**Figure 1:**
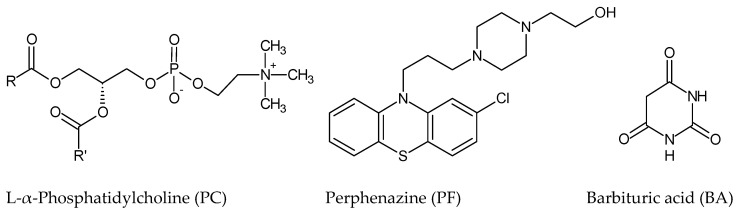
Chemical structures of compounds forming liposomes.

Central nervous system drugs can accumulate in lipid membranes and influence their structure and properties, both physicochemical and electrical. Nevertheless, there is insufficient knowledge regarding this group of drugs’ affinity to specific lipid components of membranes, which may determine their therapeutic or side effects [8]. Perphenazine is an antipsychotic drug, a phenothiazine derivative, used to treat schizophrenia and bipolar disorders [9,10]. It acts on all the levels of the central nervous system, particularly the hypothalamus [11,12]. Research has suggested that phenothiazines, including perphenazine, possess anticancer activity. The activity is mainly mediated by the drugs’ effect on the cell cycle, proliferation, or apoptosis [13,14,15]. Barbituric acid belongs to 2, 4-pyrimidine derivatives, which play an essential role in nature and pharmaceutical applications [16]. Barbiturates are depressants particularly specific to the central nervous system and can interact with other molecules through hydrogen bonds; it was proved that barbiturates form forceful hydrogen bond complexes with phosphatidylcholine [17]. Chemical structures of the CNS drugs and phosphatidylcholine are presented in Figure 1.

The effect of drugs on lipid membranes is a complex phenomenon from both the physicochemical and chemical perspectives. A drug can be affected by its interaction with the membrane components, e.g., molecular conformation or duration of the biological activity. On the other hand, the drug can also alter the membrane’s structure and properties, for instance, change its fluidity or surface charge [18,19]. In this paper, we focused on the effect of cationic (perphenazine) and anionic (barbituric acid) drug molecules on surface charge densities of zwitterionic phosphatidylcholine liposomes. Numerous membrane-mediated processes, such as drug–lipid interactions, are directly affected by pH; therefore, the influence of pH was also analyzed. Typically, the pH of extracellular fluids is 7.4, and cells regulate their internal pH at 7.0. Nevertheless, in some situations, lipid membranes are exposed to extreme pH; for example, in a mammalian stomach, membranes constantly face gastric juice with a pH between 1 and 6 [20,21]. Thus, characterizing the effect of pH on biomembranes’ electrical properties is a very important biophysical problem. When the membrane is exposed to ions, such as protons and hydroxide ions, electrostatic interactions can be altered. Biologically active molecules, including phospholipids and drugs, possess acidic or basic groups, modulating their structure and interactions [12]. Drugs are either weak bases or weak acids, and either is uncharged or charged in physiological pH. Both charged and uncharged molecules can be entrapped within the aqueous core or at the bilayer interface; however, only a fraction of uncharged drugs can pass through a lipid membrane [22]. It seems that these drugs primarily act on the polar phase of lipid bilayers. The charged moieties of phospholipid molecules (phosphate and carboxyl groups) are the main membrane binding sites for cations and the charged amine groups—for anions. The anionic form of barbituric acid predominates at pH 8.0, and the cationic form of perphenazine predominates at pH 7.0 [23]. Thus, at physiological conditions, perphenazine in an aqueous solution carries a positive and barbituric acid—a negative charge. Their binding to membranes is strongly dependent on the membranes’ lipid composition. In a pH range from 4 to 10, phosphatidylcholine molecules are zwitterionic (because the phosphate group’s negative charge is compensated by the positive charge of the choline head), whose charge distribution at the membrane interface is a function of the association of counterions. The interactions may influence surface electrical properties such as electrophoretic mobility, conductivity, surface charge density, and electrokinetic (zeta) potential [20]. The zeta potential is an essential and reliable indicator of the surface charge of membranes, and knowledge of it is crucial for the design and operation of membrane processes [24].

In the present work, binding of perphenazine and barbituric acid to phosphatidylcholine lipid membranes was investigated using the electrophoretic light scattering technique. Liposomes composed of PC were used as a model system to mimic a cell membrane and investigate interactions with selected drugs affecting the central nervous system. Except for the lipid composition and ionic strength of an electrolyte solution, pH affects the physicochemical and electrical properties of lipid membranes, such as interfacial tension or surface charge. Experimental surface charge densities of two-component liposomal membranes were calculated from electrophoretic mobility values. We focused not only on practical measurements, but also undertook a theoretical analysis of interactions existing in studied systems. We quantitatively described the equilibria between solution ions and membrane components, which allowed us to obtain numerical values of the parameters characterizing these interactions, such as membrane surface charge densities and association constants. The mathematical models were validated by comparing theoretical assumptions with experiments. To the best of our knowledge, no previous experimental and theoretical studies have been carried out on the effect of perphenazine and barbituric acid on the electrical properties of phosphatidylcholine membranes.

## 2. Theory

Charged interfaces can be described by their surface charge density and adsorbed ions. The association of solution ions to lipid membranes through Coulomb interactions is the reason why a constant charge density cannot define the resulting surface charge. If the lipid membrane is charged, the ion adsorption phenomenon can neutralize it, but if the membrane is neutral, ion adsorption can effectively charge it. The surface of membranes with zwitterionic groups is electrostatically neutral. In zwitterionic membranes, the surface charge is created by physically adsorbed ions [25]. Gouy–Chapman–Stern theory relates the electric potential and the membrane surface charge density. Using the theory, membrane surface electrostatics can be modeled quantitatively. For lipid bilayers, the fixed charges can be considered to be the *ionizable lipid’s head-groups,* whereas the adsorbed ions are electrolytes that specifically bind the head-group sites [26]. 

The four-equilibrium model quantitatively describing the interactions between zwitterionic phospholipid membrane and monovalent ions was proposed by us [27] and is presented in the work mentioned above. However, adsorption equilibria cannot always be described using four-equilibrium equations. When the total number of acidic and basic groups on a membrane surface is higher than four, it is necessary to expand the model with additional equilibria. Let us consider our systems; the liposomal membrane surface observed from the aqueous solution side has uniformly distributed surface groups of phosphatidylcholine and perphenazine/barbituric acid. Membrane surface charge density results from equilibria existing between the groups localized at the membrane surface and solution ions. Assuming that protons, hydroxide, sodium, and chloride ions adsorb at the PC/PF and PC/BA surfaces, membrane surface charge density vs. pH of the electrolyte solution can be described using six equilibrium equations.

### 2.1. A Mathematical Model Describing the Binding of Solution Ions to the Phosphatidylcholine–Perphenazine Liposomal Membrane Surface

The model assumes the existence of four equilibria associated with positively charged species of phosphatidylcholine and perphenazine with hydroxide and chloride ions. In contrast, two equilibria concern the negatively charged species of phosphatidylcholine with proton and sodium ions.

(1) $$A_{1}^{-}+H^{+}\Leftrightarrow A_{1}H$$

(2) $$A_{1}^{-}+Na^{+}\Leftrightarrow A_{1}Na$$

(3) $$B_{1}{}^{+}+OH^{-}\Leftrightarrow B_{1}OH$$

(4) $$B_{1}{}^{+}+Cl^{-}\Leftrightarrow B_{1}Cl$$

(5) $$B_{2}{}^{+}+OH^{-}\Leftrightarrow B_{2}OH$$

(6) $$B_{2}{}^{+}+Cl^{-}\Leftrightarrow B_{2}Cl$$

where$\;A_{1}^{-}$ and $B_{1}{}^{+}$ are negatively (phosphate) and positively (choline) charged groups of phosphatidylcholine, respectively. $B_{2}{}^{+}$ is the positively charged species of perphenazine. 

The association constants for electrolyte ions with charged groups located on the membrane surface are expressed as follows (the association constant for hydrogen ion to the phosphate group of phosphatidylcholine is an example below):

(7) $$K_{A_{1}H}=\frac{a_{A_{1}H}}{a_{A_{1}-\cdot \;}a_{H+}}$$

Surface concentrations (*C_PC_*, *C_PF_*) of the compounds are expressed as follows:

(8) $$a_{A_{1}-}+\;a_{A_{1}H}\;+\;a_{A_{1}Na}\;=\;C_{\mathrm{PC}}\;$$

(9) $$a_{B_{1}+}+\;a_{B_{1}OH}\;+\;a_{B_{1}Cl}\;=\;C_{\mathrm{PC}}$$

(10) $$a_{B_{2}+}+\;a_{B_{2}OH}\;+\;a_{B_{2}Cl}\;=\;C_{\mathrm{PF}}$$

(11) $$\delta \;=\;(a_{B_{1}+}\;+a_{B_{2}+}-a_{A_{1}-})\;F$$

where *δ* is the surface charge density, and *F* is the Faraday constant.

Solving the system of equations leads to the final equation (shown below) describing *PC*–*PF* membrane surface charge density:

(12) $$\frac{\delta }{F}=\;\frac{C_{PF}}{1+K_{B_{2}OH}a_{OH-}+\;K_{B_{2}Cl}a_{Cl-}}+\frac{C_{PC}}{1+K_{B_{1}OH}a_{OH-}+\;K_{B_{1}Cl}a_{Cl-}}-\frac{C_{PC}}{1+K_{A_{1}H}a_{H+}+\;K_{A_{1}Na}a_{Na+}}$$

Equation (12) needs to be simplified to a linear form at high and low *H*^+^ concentrations. Carrying out appropriate mathematical transformations leads to the obtaining of two linear equations valid for high (Equation (13)) and low (Equation (14)) concentrations of hydrogen ions.

(13) $$\frac{\delta \cdot a_{H+}}{F}=\;\left[\frac{C_{PF}}{\;1+K_{B_{2}Cl}a_{Cl-}}+\;\frac{C_{PC}}{1+\;K_{B_{1}Cl}a_{Cl-}}\right]\cdot a_{H+}-\;\left[\frac{C_{PC}K_{B_{1}OH}K_{W}}{\left(1+K_{B_{1}Cl}a_{Cl-}\right){\;}^{2}}+\frac{C_{PF}K_{B_{2}OH}K_{W}}{\left(1+K_{B_{2}Cl}a_{Cl-}\right){\;}^{2}}+\frac{C_{PC}}{K_{A_{1}H}}\right]\;$$

(14) $$\frac{\delta }{F\cdot a_{H+}}=\;-\left[\frac{C_{PC}}{1+\;K_{A_{1}Na}a_{Na+}}\right]\cdot \frac{1}{a_{H+}}+\;\left[\frac{C_{PC}}{K_{B_{1}OH\;}K_{W}}+\frac{C_{PF}}{K_{B_{2}OH\;}K_{W}}+\frac{C_{PC}K_{A_{1}H}}{{\left(1+K_{A_{1}Na}a_{Na+}\right)}^{2}}\right]$$

The application of linear regression leads to the determination of coefficients describing the linear functions. These coefficients allow us to calculate the values of parameters characterizing the association of ions at the PC/PF membrane surface (e.g., association constants). Then, Equation (12) was used to determine theoretical PC/PF membrane surface charge density values compared to experimental results to verify the model describing the system. It should be emphasized that to determine the association constants of hydroxide and chlorine ions to positively charged perphenazine, it is necessary to use the association constant data of the solution ions to the PC membrane surface, determined by our team [27].

### 2.2. A Mathematical Model Describing the Binding of Solution Ions to the Phosphatidylcholine-Barbituric Acid Liposomal Membrane Surface

The model assumes the existence of four equilibria associated with negatively charged species of phosphatidylcholine and barbituric acid with proton and sodium ions, whereas two equilibria concern the positively charged species of phosphatidylcholine with hydroxide and chloride ions.

(15) $$A_{1}^{-}+H^{+}\Leftrightarrow A_{1}H$$

(16) $$A_{1}^{-}+Na^{+}\Leftrightarrow A_{1}Na$$

(17) $$A_{2}^{-}+H^{+}\Leftrightarrow A_{2}H$$

(18) $$A_{2}^{-}+Na^{+}\Leftrightarrow A_{2}Na$$

(19) $$B_{1}{}^{+}+OH^{-}\Leftrightarrow B_{1}OH$$

(20) $$B_{1}{}^{+}+Cl^{-}\Leftrightarrow B_{1}\mathrm{Cl}$$

where$\;A_{1}^{-}$ and $B_{1}{}^{+}$ are negatively (phosphate) and positively (choline) charged groups of phosphatidylcholine, respectively. $A_{2}{}^{-}$ is the negatively charged species of barbituric acid. 

Association constants for electrolyte ions with charged groups located on the membrane surface are expressed as in the case of the phosphatidylcholine–perphenazine system (Section 2.1.).

Surface concentrations (*C_PC_*, *C_BA_*) of the compounds are expressed as follows:

(21) $$a_{A_{1}-}+\;a_{A_{1}H}\;+\;a_{A_{1}Na}\;=\;C_{PC}\;$$

(22) $$a_{B_{1}+}+\;a_{B_{1}OH}\;+\;a_{B_{1}Cl}\;=\;C_{PC}$$

(23) $$a_{A_{2}-}+\;a_{A_{2}H}\;+\;a_{A_{2}Na}\;=\;C_{BA}\;$$

(24) $$\delta \;=\;(a_{B_{1}+}-a_{A_{1}-}-a_{A_{2}-})\;F$$

where *δ* is the surface charge density, and *F* is the Faraday constant.

Solving the system of equations leads to the final equation (shown below) describing *PC*–*BA* membrane surface charge density:

(25) $$\frac{\delta }{F}=\frac{C_{PC}}{1+K_{B_{1}OH}a_{OH-}+\;K_{B_{1}Cl}a_{Cl-}}-\frac{C_{PC}}{1+K_{A_{1}H}a_{H+}+\;K_{A_{1}Na}a_{Na+}}-\frac{C_{BA}}{1+K_{A_{2}H}a_{H+}+\;K_{A_{2}Na}a_{Na+}}$$

Following the same mathematical reasoning as in Section 3.1. we obtain two linear equations correct for high (Equation (26)) and low (Equation (27)) concentrations of hydrogen ions.

(26) $$\frac{\delta \cdot a_{H+}}{F}=\;\left[\frac{C_{PC}}{1+\;K_{B_{1}Cl}a_{Cl-}}\right]\cdot a_{H+}-\;\left[\frac{C_{PC}K_{B_{1}OH}K_{W}}{\left(1+K_{B_{1}Cl}a_{Cl-}\right){\;}^{2}}+\frac{C_{PC}}{K_{A_{1}H}}+\frac{C_{BA}}{K_{A_{2}H}}\right]\;$$

(27) $$\frac{\delta }{F\cdot a_{H+}}=\;-\left[\frac{C_{PC}}{1+\;K_{A_{1}Na}a_{Na+}}+\frac{C_{BA}}{1+\;K_{A_{2}Na}a_{Na+}}\right]\cdot \frac{1}{a_{H+}}+\;\left[\frac{C_{PC}}{K_{B_{1}OH}K_{W}}+\frac{C_{PC}K_{A_{1}H}}{{\left(1+K_{A_{1}Na}a_{Na+}\right)}^{2}}+\frac{C_{BA}K_{A_{2}H}}{{\left(1+K_{A_{2}Na}a_{Na+}\right)}^{2}}\right]$$

Numeric values of the coefficients describing these linear functions were used to calculate theoretical *PC*/*BA* membrane surface charge density values compared to experimental values to verify the model describing the system. Similarly to the perphenazine-modified membrane, also in the case of determining the association constants of ions to the barbituric acid-modified membrane, it was necessary to use the data previously obtained [27].

## 3. Results and Discussion

To explore the interactions between charged drugs—perphenazine or barbituric acid and neutral lipid membranes—a series of microelectrophoretic experiments were carried out. These interactions can potentially change liposome electrical properties; therefore, membrane surface charge densities were determined using the ELS technique. All measurements were performed as a function of pH. 

### 3.1. Effect of Perphenazine on Surface Charge Densities of Neutral Phosphatidylcholine Liposomal Membranes

**Figure 2:**
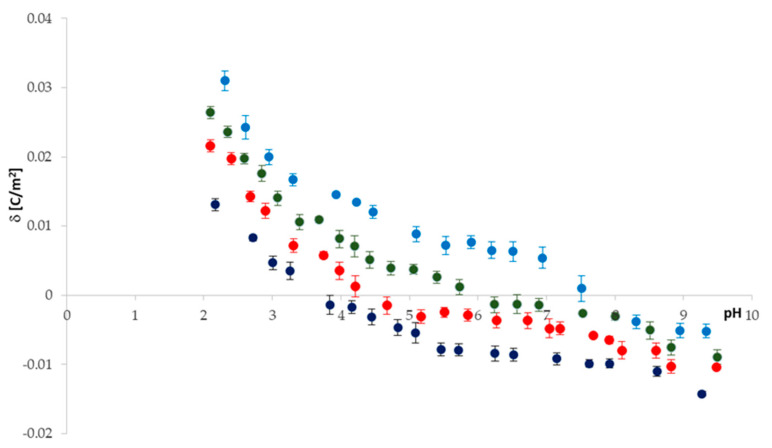
Surface charge density of pure phosphatidycholine and perphenazine-modified phosphatidylcholine membranes plotted as a function of electrolyte pH; pure PC—navy blue dots, *PC*/PF 30:1—red dots, *PC*/PF 20:1—green dots, *PC*/PF 10:1—blue dots.

Electrophoretic mobility measurements were made for pure *PC* and *PC*/*PF* 30:1; 20:1; 10:1 liposomes. The pH dependences of the surface charge densities determined from electrophoretic mobility data (Equation (28)) are given in Figure 2. Representative plots from at least three independent measurements for each of the membrane systems are presented. 

As shown in the figure, the solution pH and membrane composition have a significant effect on the surface charge of all analyzed systems. If we consider the curve obtained for a pure *PC*, we notice that the isoelectric point (IEP)—one of the most critical parameters describing variable-charge surfaces—was found to be at pH ~ 4, and the surface charge density increases (in absolute value) as the pH moves away from the IEP. As solution pH varies, differential concentrations of cations (protons and sodium ions)/anions (hydroxide and chloride ions) act as counterions to neutralize either phosphate or choline groups on *PC* lipids, thus potentially altering the surface charge [21]. For pH < 4, *PC* membranes exhibit positive surface charge density values indicating cations’ binding with the membrane surface. At pH > 4, the membranes exhibit negative surface charge density values indicating anions’ association (mainly hydroxide ions) to the membrane surface. If we consider curves obtained for perphenazine-modified liposomal membranes, we observe that the drug’s presence results in a considerable increase in surface charge densities in the whole tested pH range (pH 2–9.5). The results give evidence that perphenazine significantly influences a shift in the IEP of the *PC* membrane, from pH ~ 4 for pure *PC* to pH ~ 7.2 for 10:1 *PC*/PF membranes (Table 1). As additional data, to better clarify the obtained surface charge density results, analyzed liposomes’ electrophoretic mobilities are presented in Table 2.

The presence of perphenazine causes a change in the magnitude and even a sign of the phosphatidylcholine membrane surface charge density, and the changes are as expected. The drug is positively charged under physiological conditions, therefore its presence in the zwitterionic lipid membrane should increase its surface charge. In acidic pH, an increase in the positive charge of *PF*-treated membranes compared to the pure *PC* membrane is observed. This is caused by the shielding of the negative groups of *PC* molecules by protons coupled with exposure of the compounds’ positively charged groups and the association of chloride ions with these groups. In basic solutions, we observe more of a decrease in the negative charge of *PF*-treated membranes than pure *PC* membrane and a shift of the IEP to high pH values. The positive groups of *PC* and *PF* are shielded by hydroxide ions, while the negative groups of *PC* are exposed and are associated with sodium ions.

**Figure 3:**
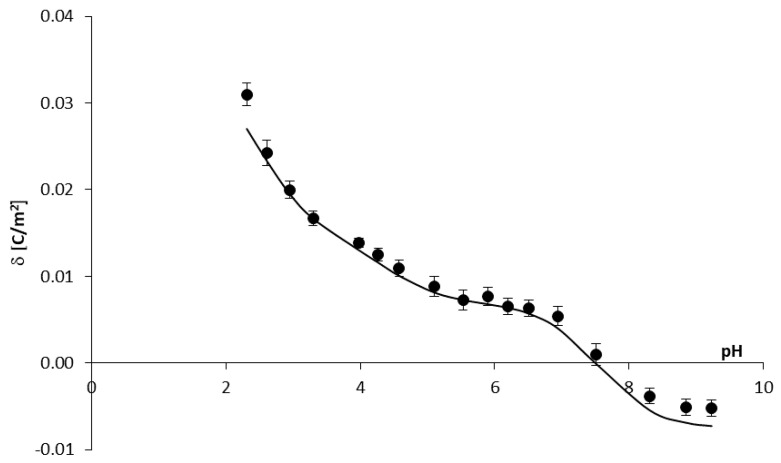
A comparison of experimental and theoretical membrane surface charge density data for the *PC*/*PF* 10:1 system (points denote experimental data and the curve—theoretical data).

Theoretical surface charge density values for the *PC*/*PF* liposomal system were determined by applying Equation (12) to the experimental data (the values for the *PC* membrane we determined previously [27]). Reported values were used to obtain numeric values of the association constants of the positive species of *PF* with *OH*^−^ and *Cl*^−^ ions from Equations (13) and (14) (Table 3). Then, after substituting them into Equation (12), theoretical data were obtained. To compare the experimental and theoretical surface charge densities vs. pH of the *PC*/*PF* 10:1 liposomal membrane, they are plotted on one graph (Figure 3). Due to a lack of legibility, data for *PC*/*PF* 30:1 and *PC*/*PF* 20:1 membranes are not included in the figure, but good fits have also been obtained for these systems.

As shown from the figure, the theoretical curve matches the experimental point in the whole pH range quite well, although it is not perfect coverage. These differences are most likely because the model describing the *PC*/*PF* membrane system does not consider interactions between the lipid and the drug, only the association of electrolyte ions to the membrane’s surface. We have attempted to extend the model (Section 2.1.) with an additional equilibrium considering a complex formation of the lipid and the drug. However, such a large number of equilibria resulted in a significant complication of the model and made it impossible to determine the searching parameters.

### 3.2. Effect of Barbituric Acid on Surface Charge Densities of Neutral Phosphatidylcholine Liposomal Membranes 

**Figure 4:**
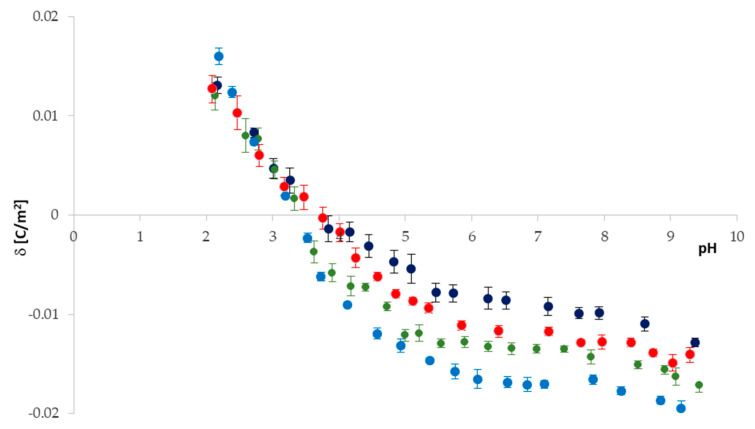
Surface charge density of pure phosphatidycholine and barbituric acid-modified phosphatidylcholine membranes, plotted as a function of electrolyte pH; pure *PC*—navy blue dots, *PC*/*BA* 30:1—red dots, *PC*/*BA* 20:1—green dots, *PC*/*BA* 10:1—blue dots.

The pH dependences of the surface charge densities for pure *PC* and *PC*/*BA* 30:1; 20:1; 10:1 liposomal membranes are presented in Figure 4. Representative plots from at least three independent measurements for each of the membrane systems are shown. 

Figure 4 and Table 4 show that similarly to the *PC*/*PF* system (Figure 2), in the case of the *PC*/*BA* system, both pH and membrane composition influence the IEP position and surface charge density of all analyzed systems. It can also be noticed that the profiles of all four curves are the same. The IEP slightly shifts towards acidic pH values as the *BA* content in the membrane increases (from pH ~ 4 for pure *PC* to pH ~ 3.2 for 10:1 *PC*/*BA* membranes). For pH < 3, there are no statistically significant changes in surface charge values of all analyzed membranes. This is because, in acidic pH, negative *PC* and *BA* species are shielded by protons, whereas positively charged *PC* groups are exposed and chloride ions bind to them. However, there is a noticeable difference between the membrane surface charge densities (including standard deviations) in the pH range between 3 and 9. Barbituric acid-modified phosphatidylcholine membranes exhibit a more negative surface charge compared to unmodified ones. These changes are more significant the higher the content of the negatively charged drug in the neutral phosphatidylcholine membrane. In basic solutions, we observe an increase in the negative charge of *BA*-treated membranes compared to the pure *PC* membrane. It is caused by the positive groups of *PC*, which are covered by hydroxide ions while the negative species of both *PC* and *BA* are exposed and are associated with sodium ions. Additionally, such as in *PC*/*PF* systems, electrophoretic mobility values of the liposomes are presented in Table 5. 

**Figure 5:**
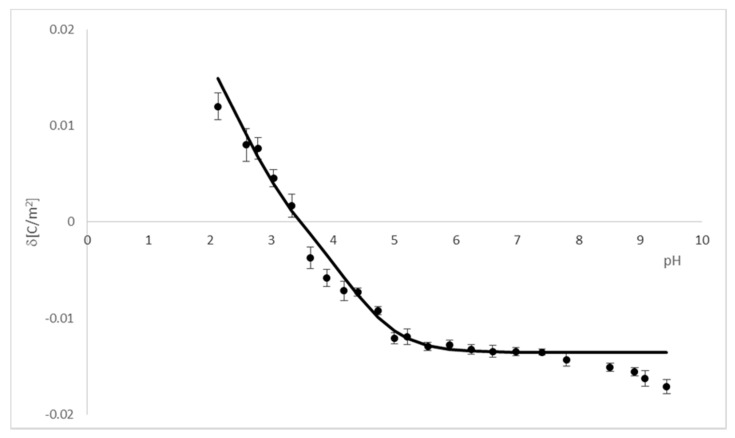
A comparison of experimental and theoretical membrane surface charge density data for the *PC*/*BA* 20:1 system (points denote experimental data and the curve—theoretical values).

The *PC*/*BA* liposomal system’s theoretical surface charge density data were determined by applying Equation (25) to the experimental data. Previously determined [27] association constants of solution ions with *PC* groups ($K_{A_{1}H},\;K_{A_{1}Na,}$ $K_{B_{1}OH},$ $K_{B_{1}Cl})$ were used to obtain association constants of the negative species of *BA* with H^+^ and Na^+^ ions ($K_{A_{2}H},K_{A_{2}Na}$) from Equations (26) and (27) (Table 6). Then, after substituting them into Equation (25), theoretical surface charge density was calculated. A comparison of experimental data and theoretical values obtained for the *PC*/*BA* 20:1 liposomal membrane is presented in Figure 5. For the graph clarity, data for the other two *BA*-modified membranes were not included in it; however, a good fit of results for these systems was also obtained.

It can be seen from the figure that the experimental points are in good agreement with the theoretical values in the range of pH 2.5–8. It is not easy to indicate precisely which interactions cause the incompatibility at other pH values; we may suppose the cause is due to the drug–lipid complex formation. Assumptions of each of the two proposed mathematical models describing interactions in the analyzed systems (Section 2.1 and Section 2.2) are, in our view, correct; however, they require an improvement. In the models, we considered the adsorption of solution ions on cationic/anionic membrane surfaces only. Undoubtedly, each of the models needs to be expanded with the equilibrium between phosphatidylcholine and perphenazine/barbituric acid. Unfortunately, the additional equilibrium complicates the models enormously because of the presence of a large number of parameters characterizing the equilibria. Therefore, it is necessary to adopt certain simplifications to reduce the number of these parameters—only then will it be possible to design all the searched values. However, despite numerous attempts, we were unable to do so.

Additionally, we measured the sizes of *PC*, *PC*/*PF*, and *PC*/*BA* liposomes (pH = 7.4). The dynamic light scattering data are presented in Table 7.

**Figure 6:**
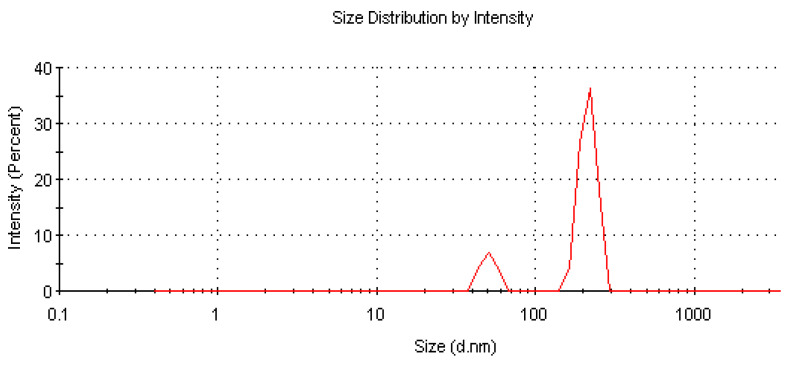
Phosphatidylcholine size distribution graph by intensity of scattered light.

It can be seen that pure *PC* liposomes and *PC* liposomes modified by both CNS drugs exhibit bimodal particle size distribution profiles and a polydispersity index (PDI) in the range between 0.294 to 0.421, indicating that analyzed liposomes are polydisperse. An example size distribution graph by the intensity of scattered light is shown in Figure 6. An increase in the particle size of liposomes containing both *PF* and *BA* compared to pure *PC* liposomes indicating that the drugs’ proportion in the formulations also influences the liposome diameter should also be noticed. Observed dependencies can be attributed to the fact that the drugs are ionized in physiological pH and interact on the lipid membrane surface.

The structure and biophysical characteristics of cell membranes may define whether and how they will respond to the drug binding. In turn, the drug’s properties may also modulate its position and conformation within biomembranes. For these reasons, investigations of drug binding to membranes become crucial to understand multi-drug resistance mechanisms or the development of undesirable side effects [28]. Our research focused on the interaction of both cationic and anionic molecules with zwitterionic lipid membranes. We selected the cationic perphenazine partly because such drugs are commonly associated with overdose and partly because they (similarly to other phenothiazines) influence electrical properties of lipid membranes. Hidalgo investigated the effect of trifluoperazine and chlorpromazine on lipid monolayers and found that binding of the drugs induces changes in surface pressure and surface potential [29]. We choose the anionic barbituric acid because it is a compound of high pharmacological importance. However, there are a lack of studies concerning barbituric acid interactions with membrane components; only data for barbiturates, such as phenobarbital or pentobarbital, can be found [30].

Membrane composition and solution pH affect its surface charge. The parameter changes are due to the modification of phosphatidylcholine membranes with the drugs acting on the central nervous system. These changes can be attributed to interactions between the membrane components and the environment and between the membrane components (lipid and drug). We want to emphasize that, to the best of our knowledge, no published study in the literature has considered interactions of the drugs with lipid membranes as a function of pH. We hope that the experimental data and determining association constants may help develop a better understanding of the influence of charged drugs on phospholipid membranes’ electrical properties. It should be emphasized that examining phenomena in which cell membranes participate and their interaction with drugs both in physiological conditions and caused or associated with particular disease states that it is crucial to exploit the molecular bases of many diseases and identify new treatment strategies.

## 4. Materials and Methods

### 4.1. Materials

L-α-phosphatidylcholine from egg yolk Type XVI-E, ≥ 99% TLC, lyophilized powder (*PC*), barbituric acid (*BA*), and perphenazine (*PF*) were purchased from Sigma-Aldrich (St. Louis, MO, USA) and used without further purification. Sodium chloride of analytical grade quality (NaCl ≥ 0.99 mass fraction purity) and chloroform (HPLC grade) were also purchased from Sigma-Aldrich. All solutions and cleaning procedures were prepared using deionized water purified to the resistance of 18.2 MΩ (HLP 5UV System, Hydrolab, Hach Company, Loveland, Co, USA) and filtered to eliminate any impurities.

### 4.2. Preparation of Liposomes Using Probe-Tip Sonication

Liposomes composed of pure *PC*, *PC*/*BA* mixed in molar ratios of 30:1, 20:1 10:1, and *PC*/*PF* mixed in molar ratios of 30:1, 20:1, 10:1, were prepared via sonication method using a probe sonicator (Techpan, Poland) shortly before measurements. The reagents (*PC*, *BA*, *PF*) were solubilized in chloroform with a ratio of 10 mg per ml of solvent. The obtained solutions were mixed in their respective molar ratios. Then, chloroform was evaporated under a gentle stream of argon to obtain a dried lipid layer, which was hydrated with 0.9% NaCl. The lipid suspension was then sonicated five times for 90 s, using an ultrasound generator. The suspensions were cooled with a mixture of dry sodium chloride and ice. The probe sonicator consisted of three main parts: a power generator, ultrasonic vibration transducer, and a sonotrode with a titanium tip. In the experiment, a tip with a 12 mm diameter and amplitude of 16 μm was used. With a maximum output power equal to 180 W, the ultrasonic generator generated vibrations at a frequency of 22 kHz. *The sonication tip released titanium into the liposome dispersion; therefore, before use, the obtained solutions were centrifuged (at 10,000 rpm for 3 min) to remove the remaining titanium and large lipid particles. Then,* the supernatant containing the liposomes was directly examined in the Zetasizer Nano ZS (Malvern Panalytical, Ltd, Malvern, UK) apparatus. The liposome sizes were evaluated by the dynamic light scattering (DLS) method at 25 °C. The size distributions were expressed as a function of the intensity of scattered light. 

### 4.3. Estimation of Experimental Surface Charge Densities from Electrophoretic Mobility Measurements

The electrophoretic light scattering technique (ELS) was used to measure liposomes’ electrophoretic mobilities as a function of pH. All measurements reported in the paper were made at room temperature (25 °C), employing a Zetasizer Nano ZS equipped with a helium–neon laser (633 nm) as a source of light, with the detection at a 90° scattering angle. Disposable folded capillary cells (Malvern DTS 1070) were used to perform measurements. A WTW InoLab pH 720 laboratory meter (WTW, *Weinheim,* Germany) was used to define the samples’ pH. Liposomes suspended in physiologic sodium chloride solution were titrated to attain the desired pH with hydrochloric acid or sodium hydroxide. pH was changed in the range 2–9.5, every ± 0.3 units. Each of the electrophoretic mobility measurements at a given pH value was carried out under identical experimental conditions (*n* = 6, each consisting of 100–200 runs with a duration of 5 s). Measurements for each liposome system (*PC*, *PC*/*PF*, *PC*/*BA*) were conducted at least three times. Experimental data are reported as means ± SD from three independent measurements. Experimental surface charge density data were determined as a function of pH by applying the following equation.

(28) $$\delta =\;\frac{\eta \cdot \mu }{d}$$

where *μ* is the electrophoretic mobility (μmcm/Vs); *η* is the viscosity of the aqueous solution (cP); and *d* is the diffuse layer thickness (m). Numerical values of *η* and *d* used were 1.4 (cP) and 7.31 × 10^−10^ (m), correspondingly.

Equation (28) is a conversion of the Smoluchowski equation for large non-conducting particles.

## Figures and Tables

**Table 1:** Effect of perphenazine on the isoelectric point (IEP) and the surface charge density of *PC* liposomes in acidic and basic solutions.

System	IEP	Surface Charge Density [10^−2^ C/m^2^]		
		pH ~ 2		pH ~ 9
*PC*	3.80 ± 0.07	1.31 ± 0.04	−1.28 ± 0.02	
*PC*/PF 30:1	4.44 ± 0.08	2.16 ± 0.05	−1.04 ± 0.02	
*PC*/PF 20:1	5.99 ± 0.13	2.64 ± 0.04	−0.89 ± 0.09	
*PC*/PF 10:1	7.23 ± 0.11	3.10 ± 0.12	−0.52 ± 0.08	

**Table 2:** Effect of perphenazine on the electrophoretic mobility of *PC* liposomes in acidic and basic solutions.

System	Electrophoretic Mobility [µmcm/Vs]	
	pH ~ 2	pH ~ 9
*PC*	0.68 ± 0.02	−0.67 ± 0.01
*PC*/PF 30:1	1.13 ± 0.03	−0.54 ± 0.02
*PC*/PF 20:1	1.38 ± 0.02	−0.46 ± 0.04
*PC*/*PF* 10:1	1.62 ± 0.07	−0.27 ± 0.03

**Table 3:** Association constants of charged groups of *PC* and *PF* with corresponding solution ions.

Compound	Association Constant [m^3^/mol]	
*PC* [27]	$K_{A_{1}H}$	7.17 × 10^2^
	$K_{A_{1}Na}$	2.30 × 10^−1^
	$K_{B_{1}OH}$	3.35 × 10^9^
	$K_{B_{1}Cl}$	7.60 × 10^−2^
*PF*	$K_{B_{2}OH}$	4.23 × 10^3^
	$K_{B_{2}Cl}$	4.01 × 10^−3^

**Table 4:** Effect of barbituric acid on the IEP and the surface charge density of *PC* liposomes in acidic and basic solutions.

System	IEP	Surface Charge Density [10^−2^ C/m^2^]	
		pH ~ 2	pH ~ 9
*PC*	3.80 ± 0.07	1.31 ± 0.04	−1.28 ± 0.02
*PC*/*BA* 30:1	3.89 ± 0.08	1.27 ± 0.14	−1.41 ± 0.06
*PC*/*BA* 20:1	3.48 ± 0.10	1.20 ± 0.09	−1.72 ± 0.10
*PC*/*BA* 10:1	3.26 ± 0.10	1.60 ± 0.12	−2.15 ± 0.07

**Table 5:** Effect of barbituric acid on the electrophoretic mobility of *PC* liposomes in acidic and basic solutions.

System	Electrophoretic Mobility [µmcm/Vs]	
	pH ~ 2	pH ~ 9
*PC*	0.68 ± 0.02	−0.67 ± 0.01
*PC*/*BA* 30:1	0.66 ± 0.08	−0.74 ± 0.03
*PC*/*BA* 20:1	0.63 ± 0.05	−0.90 ± 0.05
*PC*/*BA* 10:1	0.84 ± 0.07	−1.12 ± 0.04

**Table 6:** Association constants of charged groups of *PC* and *BA* with corresponding solution ions.

Compound	Association Constant [m^3^/mol]	
*PC* [27]	$K_{A_{1}H}$	7.17 × 10^2^
	$K_{A_{1}Na}$	2.30 × 10^−1^
	$K_{B_{1}OH}$	3.35 × 10^9^
	$K_{B_{1}Cl}$	7.60 × 10^−2^
*BA*	$K_{A_{2}H}$	2.17 × 10^1^
	$K_{A_{2}Na}$	7.30 × 10^−3^

**Table 7:** Size and polydispersity index of *PC* and *PC*/*PF*, *PC*/*BA* liposomes, pH = 7.4.

System	Liposome Size (nm)	PDI
*PC*	215.5 ± 25.8	0.404
	50.7 ± 5.5	
*PC*/*PF* 30:1	275.3 ± 16.6	0.352
	70.2 ± 18.9	
*PC*/*PF* 20:1	320.1 ± 10.6	0.384
	82.5 ± 7.6	
*PC*/*PF* 10:1	361.5 ± 11.3	0.344
	88.5 ± 8.8	
*PC*/*BA* 30:1	255.0 ± 12.1	0.392
	60.4 ± 19.4	
*PC*/*BA* 20:1	277.3 ± 8.6	0.421
	84.1 ± 9.5	
*PC*/*BA* 10:1	317.4 ± 15.7	0.294
	98.5 ± 8.4	

## Data Availability

Not applicable.

## Abbreviations

CNS	Central Nervous System
ELS	Electrophoretic light scattering
DLS	Dynamic light scattering
*PC*	L-α-phosphatidylcholine
*BA*	Barbituric acid
*PF*	Perphenazine
*δ*	Surface charge density
µ	Electrophoretic mobility
IEP	Isoelectric point
PDI	Polydispersity index
